# Prognostic value of the combined fibrinogen and prognostic nutritional index score in resectable gastric cancer

**DOI:** 10.3389/fonc.2025.1596774

**Published:** 2025-09-09

**Authors:** Shuxiang Lin, Changyue Zheng, Zehui Chen, Yulin Chen, Wei Lin

**Affiliations:** ^1^ Department of Pathology, Affiliated Hospital of Putian University, Putian, China; ^2^ School of Clinical Medicine, Fujian Medical University, Fuzhou, China; ^3^ Department of Gastrointestinal Surgery, Affiliated Hospital of Putian University, Putian, China; ^4^ Department of Anorectal Surgery, Affiliated Hospital of Putian University, Putian, China

**Keywords:** F-PNI, fibrinogen, gastric cancer, prognosis, prognostic nutritional index

## Abstract

**Objective:**

This study aimed to evaluate the prognostic value of the preoperative fibrinogen and prognostic nutritional index (PNI) combination score (F-PNI) in predicting long-term survival following radical gastrectomy in patients diagnosed with resectable gastric cancer.

**Methods:**

A retrospective cohort analysis was performed using clinicopathological and follow-up data from 491 patients who underwent radical gastrectomy for gastric cancer at a single tertiary institution between January 2012 and December 2017. Statistical analyses were conducted using SPSS version 25.0 to identify independent prognostic factors associated with five-year overall survival.

**Results:**

The optimal preoperative cut-off values were identified as 3.335 mg/L for fibrinogen and 52.7 for the PNI. The area under the receiver operating characteristic curve (AUC) for the F-PNI score was 0.633, demonstrating higher discriminative ability compared to PNI alone (AUC = 0.592) and fibrinogen alone (AUC = 0.587). Kaplan–Meier survival analysis showed five-year survival rates as follows: low vs. high fibrinogen levels, 72.9% vs. 56.4%; high vs. low PNI, 82.6% vs. 58.0%; and F-PNI score 0, 1, and 2, 87.8%, 66.9%, and 50.0%, respectively. Both univariate and multivariate Cox proportional hazards models identified a high F-PNI score, advanced age, pathological TNM stage IIIA–IIIC, and vascular invasion as independent prognostic factors for five-year survival. The F-PNI score demonstrated superior prognostic performance compared to either fibrinogen or PNI alone.

**Conclusion:**

The F-PNI combination score is an independent prognostic marker for long-term survival in patients undergoing radical resection for resectable gastric cancer. Its enhanced predictive accuracy relative to fibrinogen or PNI individually suggests potential utility in preoperative prognostic stratification.

## 1 Introduction

Gastric cancer (GC) is a prevalent malignancy, ranking fifth in global cancer incidence and third in cancer-related mortality worldwide (1). In its early stages, GC is frequently asymptomatic or presents with nonspecific symptoms, leading to a high proportion of cases being diagnosed at an advanced stage. Although radical surgery combined with postoperative adjuvant chemotherapy has improved survival outcomes, a substantial number of patients experience postoperative recurrence, metastasis, or tumor-related mortality, resulting in suboptimal long-term prognosis (2).

Current prognostic evaluation for GC primarily relies on the tumor-node-metastasis (TNM) staging system, which assesses key pathological characteristics, including depth of tumor invasion (T), lymph node involvement (N), and the presence of distant metastasis (M). However, the TNM system alone does not fully account for the biological heterogeneity of GC, as various additional factors influence long-term outcomes. Previous studies have demonstrated that systemic inflammation, hypercoagulability, and malnutrition are significantly associated with unfavorable prognosis following radical gastrectomy (3).

In recent years, several prognostic scoring systems incorporating hematological biomarkers have been developed to improve the accuracy of survival prediction in GC. These systems evaluate parameters such as systemic inflammatory response, coagulation status, and nutritional condition, all of which have been shown to correlate with clinical outcomes. Given that these biomarkers are routinely assessed through accessible, cost-effective blood tests, they hold considerable clinical value in prognostic stratification for patients with GC.

Fibrinogen (Fib), is widely recognized as a marker of systemic inflammation and hypercoagulability. Multiple studies have confirmed a strong association between elevated Fib levels and poor oncologic outcomes (4). The Prognostic Nutritional Index (PNI), calculated using serum albumin concentration and peripheral lymphocyte count, is a well-established indicator of nutritional and immune status.

Despite their individual prognostic relevance, the utility of single biomarkers may be constrained by limited sensitivity and specificity. While Fib predominantly reflects systemic inflammation and coagulation, PNI more accurately reflects nutritional status. Recent evidence supports the integration of multiple biomarkers to enhance predictive performance. Consequently, this study evaluates the combined prognostic value of preoperative Fib and PNI—the F-PNI score, in patients undergoing radical gastrectomy for resectable GC, with the aim of improving long-term survival prediction.

Given the substantial heterogeneity in physiological and pathological characteristics among patients with malignant disease, personalized therapeutic strategies are critical to optimizing clinical outcomes. Identifying high-risk subgroups and clinically relevant prognostic markers is essential for guiding individualized treatment planning in GC.

## 2 Data and methods

### 2.1 Research participants

A retrospective analysis was conducted on patients diagnosed with resectable GC who underwent radical gastrectomy at the Affiliated Hospital of Putian University between January 2012 and December 2017. Comprehensive clinical and pathological data were collected for analysis.

The inclusion criteria were as follows: (1) histopathologically confirmed diagnosis of GC without evidence of distant metastasis detected preoperatively or intraoperatively; (2) no history of neoadjuvant chemotherapy or neoadjuvant chemoradiotherapy; (3) postoperative pathological confirmation of R0 resection; (4) availability of complete preoperative hematological examination results, including Fib levels, serum albumin values, and peripheral blood lymphocyte count; and (5) completeness of postoperative pathological data and follow-up information.

The exclusion criteria included the following: (1) presence of distant metastasis or primary malignant tumors in other internal organs; (2) acute inflammatory diseases, liver cirrhosis, chronic renal failure, autoimmune diseases, or hematopoietic system disorders; (3) incomplete clinical data or loss to follow-up. (4) patients with acute inflammatory diseases, liver cirrhosis, chronic renal failure, autoimmune diseases, hematologic disorders, or other primary malignancies in different organs.

A total of 491 patients met the inclusion criteria and were enrolled in the study. The surgical procedure and extent of lymphadenectomy were performed in accordance with the Japanese Gastric Cancer Treatment Guidelines. Pathological staging was conducted based on the eighth edition of the TNM classification system, jointly established by the Union for International Cancer Control (UICC) and the American Joint Committee on Cancer (AJCC).

Ethical approval for the study was obtained from the Ethics Committee of the Affiliated Hospital of Putian College. All procedures were conducted in accordance with the principles outlined in the Declaration of Helsinki and its subsequent amendments. As this was a retrospective study with anonymized data and no identifiable patient information, the requirement for informed consent was waived.

### 2.2 Data and general information

General patient information, including age and sex, was obtained from medical records. Tumor-related pathological data included maximum tumor diameter, anatomical location, histological differentiation, presence of vascular and perineural invasion, depth of invasion (T stage), regional lymph node metastasis (N stage), and overall TNM classification. Clinical parameters comprised preoperative Fib levels, serum albumin concentration, peripheral blood lymphocyte count, and whether postoperative adjuvant chemotherapy was administered.

Fasting peripheral blood samples were collected within one week prior to surgery for laboratory analysis. The PNI was calculated using the following formula:


$$\begin{array}{c} PNI=\text{serum albumin}\left(g/L\right)+5\times \\ \text{total peripheral blood lymphocyte count}\left(\times 10^{9}/L\right). \end{array}$$

Receiver operating characteristic (ROC) curve analysis was employed to determine the optimal cut-off values for Fib and PNI, allowing for the stratification of patients into prognostic subgroups. The F-PNI was defined according to the following criteria: Patients with low Fib and high PNI were classified as F-PNI = 0. Those with either high Fib and high PNI or low fibrinogen and low PNI were assigned an F-PNI score of 1. Patients exhibiting high Fib and low PNI were designated as F-PNI = 2.

### 2.3 Follow-up data

Patients were followed up regularly through outpatient clinic visits, hospital admissions, or telephone consultations. During the first two years postoperatively, follow-up assessments were conducted every three months. From the third to the fifth postoperative year, evaluations occurred at six-month intervals. After five years, annual follow-up visits were scheduled.

Each follow-up included routine hematological and biochemical testing, measurement of gastric tumor markers, imaging studies such as abdominal ultrasonography or computed tomography (CT), and annual upper gastrointestinal endoscopy. Survival time was defined as the interval from the date of surgery to either the date of death or the most recent follow-up contact. As of December 2022, the median follow-up duration was 48 months. The five-year survival time and overall survival rate were calculated accordingly.

### 2.4 Statistical methods

All statistical analyses were performed using IBM SPSS Statistics version 25.0. Continuous variables are presented as mean ± standard deviation, and intergroup comparisons were conducted using the independent samples *t*-test. Categorical variables were analyzed using the chi-squared test.

ROC curve analysis was used to determine the optimal cut-off values for preoperative fibrinogen and PNI. The prognostic performance of each variable was assessed accordingly. Survival outcomes were evaluated using the Kaplan–Meier method, with survival curves compared using the log-rank test. Univariate and multivariate Cox proportional hazards regression analyses were performed to identify independent prognostic factors associated with five-year overall survival following radical gastrectomy for resectable GC. A *p*-value of < 0.05 was considered statistically significant.

## 3 Result

### 3.1 General clinical and pathological data

Among the 491 patients included in the study, a total of 168 deaths were recorded during the follow-up period. The five-year overall survival rate was 65.8%, with a median follow-up duration of 50 months. GC was more frequently diagnosed in male patients, with a male-to-female ratio of approximately 3.8:1. The majority of cases occurred in older adults, and tumors were most commonly located in the cardia. A considerable proportion of patients received postoperative adjuvant chemotherapy.

Pathological evaluation revealed that tumors with a maximum diameter of less than 5 cm were relatively common. The pathological T4 stage was the most frequently observed, and regional lymph node metastasis was identified in approximately 50% of cases. Pathological TNM stages II and III were predominant, comprising 336 patients (68.5%). Most tumors were classified as poorly or moderately differentiated, accounting for approximately 88.2% of the cohort. Perineural invasion was present in 18.7% of patients, while vascular invasion was observed in 18.0% (see **Table 1**).

**Table 1:** Composition of basic clinicopathological data.

Study variables	Number of cases (Percentage composition)
Age (years)	
<60	204(41.5%)
≥60	287(58.5%)
Gender	
Male	383(78.0%)
Female	108(22.0%)
Tumor location	
Gastroesophageal junction	248(50.5%)
Gastric body	109(22.2%)
Gastric fundus	7(1.4%)
Gastric angle	40(8.1%)
Gastric antrum	87(17.7%)
Tumor size(cm)	
< 5	313(63.7%)
≥5	178(36.3%)
Pathological T staging	
pT1	108(22%)
pT2	65(13.2%)
pT3	19(3.9%)
pT4	299(60.9%)
Pathological N staging	
pN0	221(45%)
pN1	70(14.3%)
pN2	92(18.7%)
pN3	108(22%)
TNM staging	
IA-IB	155(31.6%)
IIA-IIB	99(20.2%)
IIIA-IIIC	237(48.3%)
Tumor differentiation	
High	58(11.8%)
Medium	197(40.1%)
Low	236(48.1%)
Neural infiltration	
No	399(81.3%)
Yes	92(18.7%)
Vascular infiltration	
No	400(81.5%)
Yes	91(18.5%)
Postoperative chemotherapy	
No	205(41.8%)
Yes	286(58.2%)

These findings suggest that the majority of patients were diagnosed at an advanced stage of GC.

### 3.2 Determination of optimal cut-off values for preoperative Fib, PNI, and their correlation with clinical characteristics

**Figure 1:**
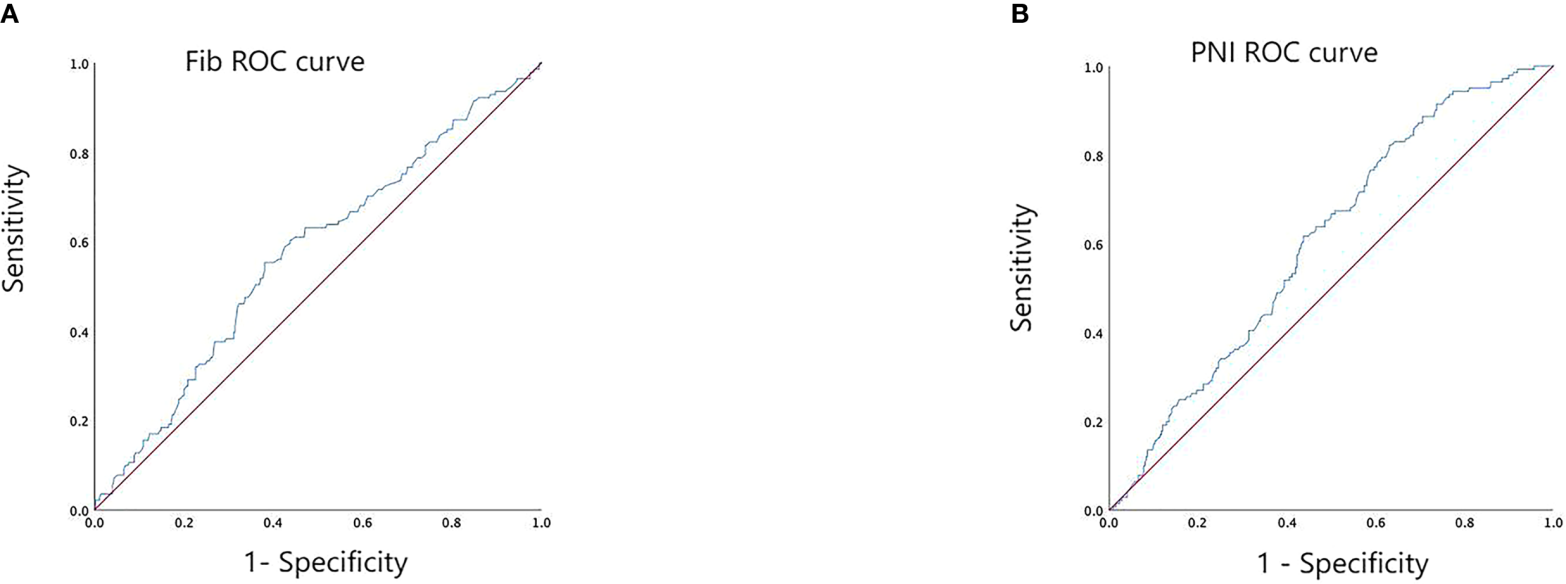
ROC curve analysis for determining the optimal cut-off values of preoperative Fib **(A)** and PNI **(B)**.

ROC curve analysis was performed using preoperative Fib and PNI values to evaluate their predictive performance for five-year overall survival in the cohort of 491 patients. The area under the ROC curve (AUC) for Fib was 0.572 (95% confidence interval [CI]: 0.516 – 0.628, *p* = 0.012), while the AUC for PNI was 0.604 (95% CI: 0.552 – 0.606, *p* < 0.001) (see **Figure 1**). The optimal cut-off values, determined based on the maximum Youden’s index, were 3.335 mg/L for Fib (sensitivity: 0.553; specificity: 0.380) and 52.7 for PNI (sensitivity: 0.823; specificity: 0.369). Based on these thresholds, patients were stratified into low Fib (≤ 3.335 mg/L) and high Fib (> 3.335 mg/L) groups, as well as low PNI (≤ 52.7) and high PNI (> 52.7) groups.

Comparative analysis between the low and high Fib groups showed that patients in the high Fib group were more likely to present with larger tumors, more advanced pathological T, N, and TNM stages, lower histological differentiation, and an increased likelihood of receiving postoperative adjuvant chemotherapy. These differences were statistically significant (*p* < 0.05). No significant differences were observed between the groups with respect to age, sex, tumor location, or the presence of perineural or vascular invasion (*p* > 0.05).

Similarly, comparison between the low and high PNI groups revealed that patients in the low PNI group were more likely to be older, male, and have tumors exceeding 5 cm in diameter. These patients more frequently presented with tumors of intermediate to advanced pathological T, N, and TNM stages, poorer differentiation, and a higher incidence of perineural and vascular invasion. A greater proportion of patients in the low PNI group also received postoperative adjuvant chemotherapy. These differences were statistically significant (*p* < 0.05). No significant association was found between PNI group and tumor location (*p* > 0.05) (see **Table 2**).

**Table 2:** Relationship between preoperative Fib and PNI and clinicopathological features of gastric cancer patients.

Study variables	Fib		P value	PNI		P value
	Low level group	High level group		High PNI	Low PNI	
Age (years)			0.23			0.001
<60	123(43.9%)	81(38.4%)		82(47.1%)	122(36.3%)	
≥60	157(56.1%)	130(61.6%)		73(52.9%)	214(63.7%)	
Gender			0.913			0.014
Male	219(78.2%)	164(77.7%)		110(71.0%)	273(81.3%)	
Female	61(21.8%)	47(22.3%)		45(29.0%)	63(18.7%)	
Tumor location			0.284			0.367
Gastroesophageal junction	139(49.6%)	109(51.7%)		69(44.5%)	179(53.3%)	
Gastric body	63(22.5%)	46(21.8%)		40(25.8%)	69(20.5%)	
Gastric fundus	4(1.4%)	3(1.4%)		2(1.3%)	5(1.5%)	
Gastric angle	29(10.4%)	11(5.2%)		16(10.3%)	24(7.1%)	
Gastric antrum	45(16.1%)	42(19.9%)		28(18.1%)	59(17.6%)	
Tumor size(cm)			<0.001			<0.001
< 5	207(73.9%)	106(50.2%)		122(78.7%)	191(56.8%)	
≥5	73(26.1%)	105(49.8%)		33(21.3%)	145(43.2%)	
Pathological T staging			<0.001			<0.001
pT1	80(28.6%)	28(13.3%)		52(33.5%)	56(16.7%)	
pT2	46(16.4%)	19(9.0%)		31(20%)	34(10.1%)	
pT3	11(3.9%)	8(3.8%)		5(3.2%)	14(4.2%)	
pT4	143(51.1%)	156(73.9%)		67(43.3%)	232(69.1%)	
Pathological N staging			<0.001			<0.001
pN0	150(53.6%)	71(33.6%)		91(58.7%)	130(38.7%)	
pN1	30(10.7%)	40(19.0%)		24(15.5%)	46(13.7%)	
pN2	50(17.9%)	42(19.9%)		21(13.5%)	71(21.1%)	
pN3	50(17.9%)	58(27.5%)		19 (12.3%)	89(26.5%)	
TNM staging			<0.001			<0.001
IA-IB	115(41.1%)	40(19.0%)		74(47.7%)	81(24.1%)	
IIA-IIB	56(20.0%)	43(20.4%)		31(20.0%)	68(20.2%)	
IIIA-IIIC	109(38.9%)	128(60.7%)		50(32.3%)	187(55.7%)	
Tumor differentiation			0.003			0.002
High	41(14.6%)	17(8.1%)		28(18.1%)	30(8.9%)	
Medium	122(43.6%)	75(35.5%)		67(43.2%)	130(38.7%)	
Low	117(41.8%)	119(56.4%)		60(38.7%)	176(52.4%)	
Neural infiltration			0.728			0.025
No	229(81.8%)	170(80.6%)		135(87.1%)	264(78.6%)	
Yes	51(18.2%)	41(19.4%)		20(12.9%)	72(21.4%)	
Vascular infiltration			0.197			0.008
No	234(83.6%)	166(78.7%)		137(88.4%)	263(78.3%)	
Yes	46(16.4%)	45(21.3%)		18(11.6%)	73(21.7%)	
Postoperative chemotherapy			<0.001			0.01
No	140(50.0%)	65(30.8%)		78(50.3%)	127(37.8%)	
Yes	140(50.0%)	146(69.2%)		77(49.7%)	209(62.2%)	

### 3.3 The value of Fib, PNI, and F-PNI in prognostic assessment

The F-PNI score was calculated based on preoperative Fib and PNI levels. A score of 0 was assigned to patients with low Fib and high PNI levels. A score of 1 was assigned when both Fib and PNI levels were either high or low. A score of 2 was assigned to patients with high Fib and low PNI levels. Based on this classification, the study cohort was divided into three groups: 98 patients (F-PNI = 0), 239 patients (F-PNI = 1), and 154 patients (F-PNI = 2).

**Figure 2:**
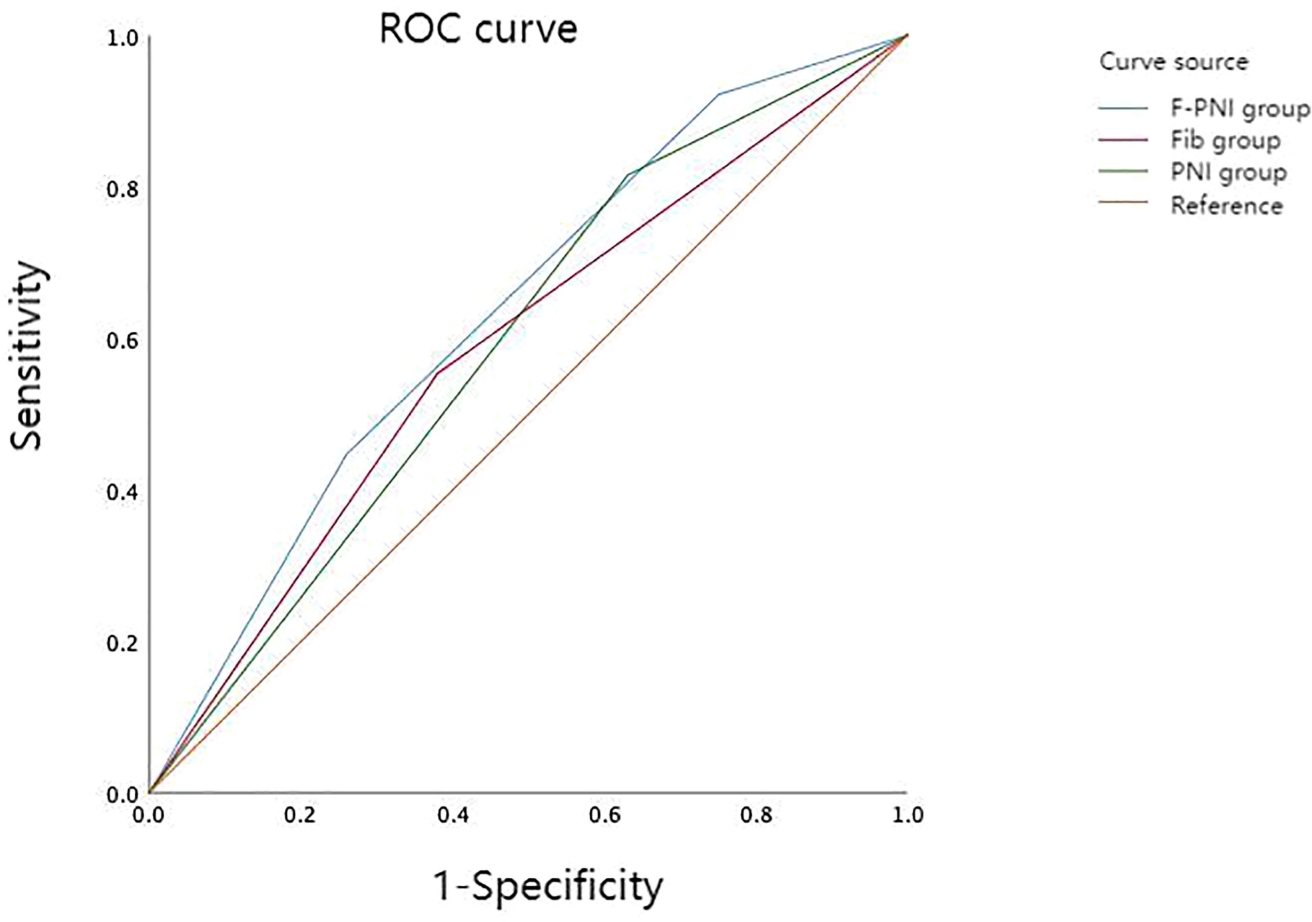
Comparison of predictive performance among prognostic indicators for overall survival in GC. AUC for F-PNI = 0.633; PNI = 0.592; Fib = 0.587.

ROC curve analysis was used to compare the prognostic performance of Fib, PNI, and the combined F-PNI score. The AUC for the F-PNI score was 0.633, which was greater than that of PNI (AUC = 0.592) and Fib (AUC = 0.587), as shown in **Table 3**. These results suggest that the F-PNI score offers superior prognostic discrimination for long-term survival following radical gastrectomy compared to either biomarker alone. The comparative ROC curves are shown in **Figure 2**.

**Table 3:** Area under the ROC curve of different predictors.

Test outcome variables	Area under curve	95% confidence interval	P value
F-PNI composite score	0.633	0.581-0.686	<0.001
PNI	0.592	0.539-0.646	0.001
Fib	0.587	0.531-0.643	0.003

The association between Fib, PNI, and F-PNI scores and five-year overall survival was further examined using Kaplan–Meier survival analysis. The overall five-year survival rate among the 491 patients was 65.8%.

**Figure 3:**
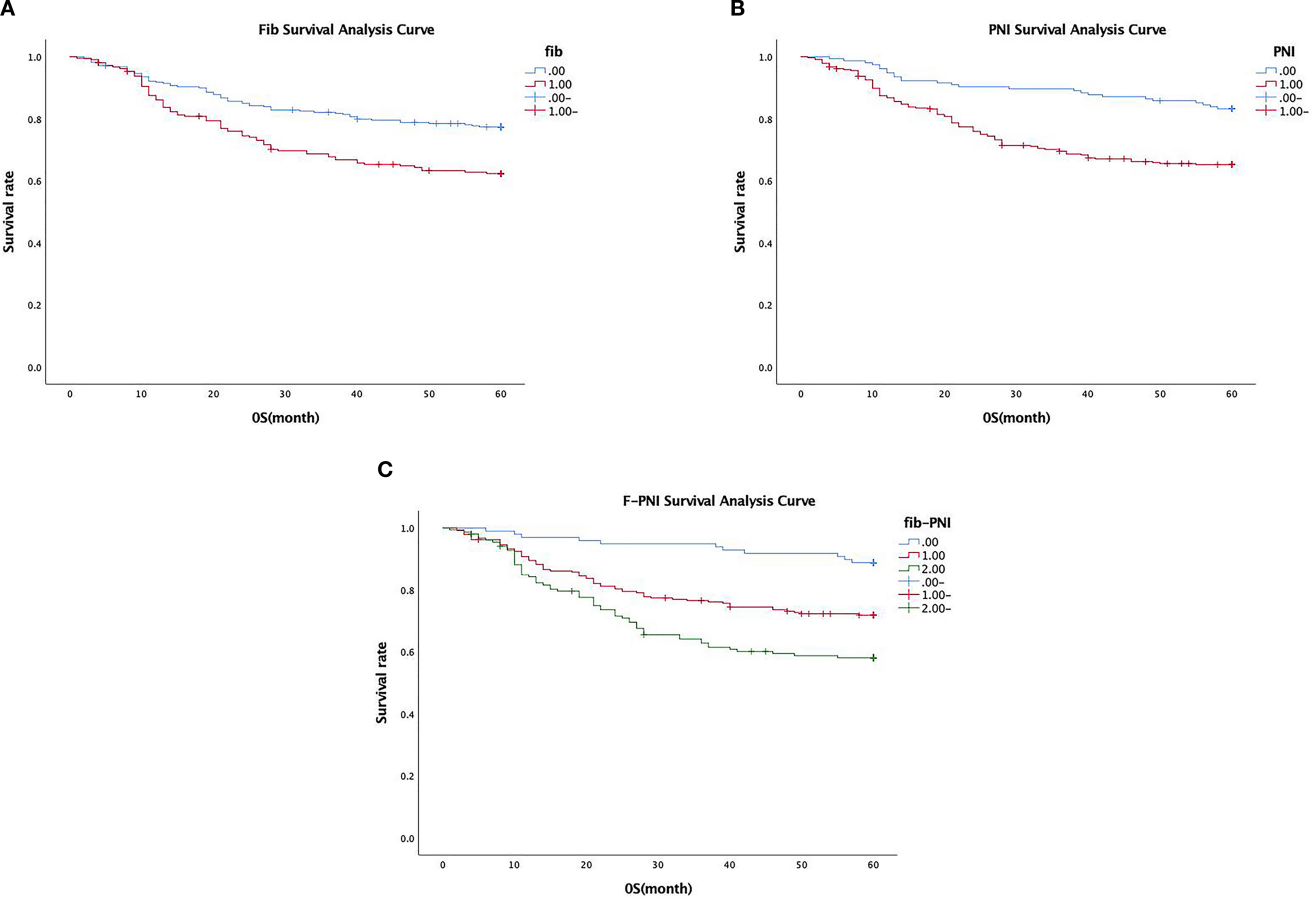
Association between preoperative Fib **(A)**, PNI **(B)**, and combined F-PNI **(C)** scores and five-year survival in GC. All differences were statistically significant (*p* < 0.01).

Patients in the low Fib group demonstrated a significantly higher five-year survival rate (72.9%) compared to those in the high Fib group (56.4%) (*p* < 0.001) (see **Figure 3A**). Similarly, survival outcomes were significantly better in the high PNI group (82.6%) compared to the low PNI group (58.0%) (*p* < 0.001) (see **Figure 3B**). A strong association was also observed between the F-PNI score and long-term survival. The five-year survival rates were 87.8% for patients with F-PNI = 0, 66.9% for those with F-PNI = 1, and 50.0% for those with F-PNI = 2 (*p* < 0.001), indicating statistically significant differences among the three groups (see **Figure 3C**).

Detailed five-year survival data stratified by Fib, PNI, and F-PNI scores are presented in **Table 4**.

**Table 4:** Survival analysis on the 5-year survival rate of gastric cancer patients.

Study variables	5-year survival rate	P value
Overall	65.80%	
Fib		<0.001
Low Fib group	72.90%	
High Fib group	56.40%	
PNI		<0.001
PNI high level group	82.60%	
PNI low level group	58.00%	
F-PNI		<0.001
F-PNI=0	87.80%	
F-PNI=1	66.90%	
F-PNI=2	50.00%	

### 3.4 Correlation between the composite F-PNI score and clinicopathological characteristics

The composite F-PNI score demonstrated significant associations with several clinicopathological parameters. Higher F-PNI scores were observed in a greater proportion of older patients and were associated with larger tumor diameters and more advanced pathological staging, including T stage, N stage, and overall TNM classification. An increase in F-PNI score also corresponded with poorer tumor differentiation, a higher incidence of vascular invasion, and a greater likelihood of receiving postoperative adjuvant chemotherapy. These differences among the F-PNI score groups were statistically significant (*p* < 0.05).

In contrast, no statistically significant associations were identified between the F-PNI score and patient sex, tumor location, or the presence of perineural invasion (*p* > 0.05) (see **Table 5**).

**Table 5:** The relationship between F-PNI analysis and clinicopathological features of gastric cancer patients.

Study variables	F-PNI				P value	
	0	1	2			
Age (years)				0.007		
<60	51(52.0%)	103(43.1%)	50(32.5%)		
≥60	47(48.0%)	136(56.9%)	104(67.5%)		
Gender				0.263		
Male	71(72.4%)	187(78.2%)	125(81.2%)		
Female	27(27.6%)	52(21.8%)	29(18.8%)		
Tumor location				0.223		
Gastroesophageal junction	42(42.9%)	124(51.9%)	82(53.2%)		
Gastric body	24(24.5%)	55(23.0%)	30(19.5%)		
Gastric fundus	1(1.0%)	4(1.7%)	2(1.3%)		
Gastric angle	15(15.3%)	15(6.3%)	10(6.5%)		
Gastric antrum	16(16.3%)	41(17.2%)	30(19.5%)		
Tumor size(cm)				<0.001		
< 5	85(86.7%)	159(66.5%)	69(44.8%)		
≥5	13(13.3%)	80(33.5%)	85(55.2%)		
Pathological T staging				<0.001		
pT1	39(39.8%)	54(22.6%)	15(9.7%)		
pT2	20(20.4%)	37(15.5%)	8(5.2%)		
pT3	3(3.1%)	10(4.2%)	6(3.9%)		
pT4	36(36.7%)	138(57.7%)	125(81.2%)		
Pathological N staging				<0.001		
pN0	65(66.3%)	111(46.4%)	45(29.2%)		
pN1	12(12.2%)	30(12.6%)	28(18.2%)		
pN2	13(13.3%)	45(18.8%)	34(22.1%)		
pN3	8(8.2%)	53(22.2%)	47(30.5%)		
TNM staging				<0.001		
IA-IB	54(55.1%)	81(33.9%)	20(13.0%)		
IIA-IIB	20(20.4%)	47(19.7%)	32(20.8%)		
IIIA-IIIC	24(24.5%)	111(46.4%)	102(66.2%)		
Tumor differentiation				<0.001		
High	19(19.4%)	31(13.0%)	8(5.2%)		
Medium	45(45.9%)	99(41.4%)	53(34.4%)		
Low	34(34.7%)	109(45.6%)	93(60.4%)		
Neural infiltration				0.217		
No	85(86.7%)	194(81.2%)	120(77.9%)		
Yes	13(13.3%)	45(18.8%)	34(22.1%)		
Vascular infiltration				0.017		
No	89(90.8%)	193(80.8%)	118(76.6%)		
Yes	9(9.2%)	46(19.2%)	36(23.4%)		
Postoperative chemotherapy				<0.001		
No	58(59.2%)	102(42.7%)	45(29.2%)		
Yes	40(40.8%)	137(57.3%)	109(70.8%)		

### 3.5 Univariate and multivariate Cox regression analysis of factors influencing the 5-year survival rate

Univariate and multivariate Cox proportional hazards regression analyses were conducted to evaluate factors associated with five-year overall survival, incorporating Fib, PNI, the combined F-PNI score, and other relevant clinicopathological variables. The multivariate analysis identified the F-PNI score as an independent prognostic factor. Using F-PNI = 0 as the reference category, patients with F-PNI = 1 had a significantly increased risk of mortality (hazard ratio [HR] 2.554, 95% confidence interval [CI]: 1.275 – 5.117, *p* = 0.008), and those with F-PNI = 2 had an even greater risk (HR 2.736, 95% CI: 1.357 – 5.518, *p* = 0.005).

Additional independent prognostic factors included advanced age (HR 1.664, 95% CI: 1.177 – 2.353, *p* = 0.004), pathological TNM stage IIIA–IIIC (HR 13.713, 95% CI: 6.312 – 29.790, *p* < 0.001), and the presence of vascular invasion (HR 1.499, 95% CI: 1.062 – 2.117, *p* = 0.021).

These findings support the superior prognostic value of the combined F-PNI score over Fib and PNI as individual biomarkers in predicting long-term survival following radical gastrectomy (see **Table 6**).

**Table 6:** Univariate analysis and Multivariate analysis on the 5-year survival rate of gastric cancer patients.

Study variables	Univariate analysis		Multivariate analysis	
	HR(95%CI)	P value	HR(95%CI)	P value
Fib				
Low Fib group	1		1	
High Fib group	1.820(1.319-2.511)	<0.001	1.262(0.832-1.692)	0.261
PNI				
PNI high level group	1		1	
PNI low level group	2.814(1.818-4.355)	<0.001	2.07(1.386-2.754)	0.15
F-PNI				
F-PNI=0	1		1	
F-PNI=1	3.826(1.914-7.648)	<0.001	2.554(1.275-5.117)	0.008
F-PNI=2	6.078(3.031-12.185)	<0.001	2.736(1.357-5.518)	0.005
Age (years)				
<60	1		1	
≥60	1.619(1.149-2.281)	0.006	1.664(1.177-2.353)	0.004
Gender				
Male	1		1	
Female	0.940(0.636-1.389)	0.756	0.056(-0.724-0.836)	0.812
Tumor location				
Gastroesophageal junction	1		1	
Gastric body	0.686(0.445-1.056)	0.087	1.543(1.038-2.048)	0.214
Gastric fundus	1.313(0.415-4.153)	0.643	2.13(1.908-2.352)	0.144
Gastric angle	0.600(0.302-1.193)	0.145	0.002(-0.012-0.016)	0.969
Gastric antrum	0.819(0.525-1.279)	0.380	1.435(1.354-1.516)	0.231
Tumor size(cm)				
< 5	1		1	
≥5	2.877(2.081-3.979)	<0.001	0.673(0.036-1.31)	0.412
Pathological T staging				
pT1	1		1	
pT2	2.533(0.715-8.975)	0.15	2.008(1.788-2.228)	0.156
pT3	7.585(2.037-28.249)	0.003	0.753(0.621-0.885)	0.386
pT4	16.252(6.010-43.947)	<0.001	0.048(0.009-0.087)	0.827
Pathological N staging				
pN0	1		1	
pN1	5.645(2.666-11.952)	<0.001	9.437(8.987-9.887)	0.002
pN2	12.531(6.477-24.244)	<0.001	18.245(18.102-18.388)	<0.001
pN3	25.89(13.724-48.84)	<0.001	13.113(12.926-13.3)	<0.001
TNM staging				
IA-IB	1		1	
IIA-IIB	2.277(0.867-5.983)	0.095	1.869(0.709-4.930)	0.206
IIIA-IIIC	18.162(8.486-38.873)	<0.001	13.713(6.312-29.790)	<0.001
Tumor differentiation				
High	1		1	
Medium	3.592(1.292-9.987)	0.014	0.016(-0.102-0.134)	0.899
Low	7.796(2.870-21.180)	<0.001	0.023(-0.378-0.424)	0.88
Neural infiltration				
No	1		1	
Yes	1.806(1.261-2.586)	<0.001	1.499(1.062-2.117)	0.021
Vascular infiltration				
No	1		1	
Yes	2.864(2.041-4.019)	<0.001	1.303(0.488-2.118)	0.254
Postoperative chemotherapy				
No	1		1	
Yes	2.091(1.460-2.995)	<0.001	1.130(0.548-1.712)	0.288

## 4 Discussion

Advancements in the comprehensive management of GC in recent years have contributed to improved survival rates and enhanced quality of life for patients (5). Despite these gains, GC continues to be associated with poor prognosis, largely due to late-stage diagnosis in a substantial proportion of cases. In the present study, most patients were diagnosed at advanced stages, consistent with the ongoing clinical challenge of early detection and the associated negative impact on long-term outcomes.

Various prognostic scoring systems based on hematological biomarkers have recently demonstrated potential in predicting survival outcomes in GC. These systems typically assess clinical features such as hypercoagulability, nutritional status, and systemic inflammation—factors that have been shown to influence tumor progression and overall prognosis (6). In this study, Fib and the PNI were selected as representative biomarkers reflecting these physiological domains.

Both Fib and PNI are derived from routine blood tests, making them simple, accessible, and cost-effective tools in clinical practice. Because these parameters can be obtained through standard preoperative testing, they hold considerable clinical value for the prognostic evaluation of patients with GC. The integration of such streamlined screening measures facilitates the early identification of high-risk patients and supports the development of personalized, risk-adapted treatment strategies. These individualized approaches may contribute to improved survival outcomes and more efficient allocation of clinical resources.

### 4.1 Impact of Fib on the five-year post-operative survival rate

Fib is a glycoprotein synthesized by the liver that plays a central role in the coagulation cascade and serves as a biomarker of hypercoagulability (7). Previous studies have established strong associations between elevated Fib levels and tumorigenesis, angiogenesis, and metastasis. Fib contributes to tumor progression through its conversion into fibrin, which promotes angiogenesis and facilitates tumor invasion and metastasis (8).

Fib plays a key role in the coagulation cascade and has been identified as a relevant biomarker in oncologic prognostication. Numerous studies have demonstrated that elevated plasma Fib levels are associated with tumor progression, local invasion, distant metastasis, and poor clinical outcomes in patients with GC (9, 10). Increased Fib concentrations are often indicative of more advanced tumor stages and are linked to reduced survival durations.

Ding et al. reported that malignant tumors are frequently accompanied by hypercoagulable states and enhanced conversion of Fib to fibrin, a process that contributes to tumor proliferation, angiogenesis, and metastatic spread, thereby resulting in worse prognostic outcomes (10). In the present study, both univariate and multivariate analyses revealed that patients with elevated preoperative Fib levels had significantly lower five-year survival rates and shorter overall survival compared to those with lower Fib levels. These findings reinforce the role of Fib as an independent prognostic risk factor in resectable GC and are consistent with previous literature.

### 4.2 Impact of PNI on the five-year post-surgery survival rate

PNI, derived from serum albumin concentration and peripheral lymphocyte count, is a well-established indicator for assessing preoperative immune competence and nutritional status in patients undergoing gastrointestinal surgery (11). Prior research has consistently demonstrated that nutritional and immune status at the time of diagnosis significantly influences clinical outcomes and overall prognosis in patients with malignant tumors (12). Reduced PNI values are frequently indicative of malnutrition and immunosuppression, both of which have been associated with poor survival outcomes.

In recent years, PNI has gained increasing recognition as a simple, cost-effective, and clinically relevant prognostic biomarker across various malignancies (13). Malignant tumors often increase systemic metabolic demands, leading to enhanced protein catabolism and nutritional depletion. In the present study, PNI was identified as an independent prognostic factor affecting long-term survival in patients with resectable GC. Patients with lower PNI values exhibited shorter survival durations, decreased five-year survival rates, and poorer overall prognoses.

Multiple studies have validated the prognostic utility of PNI in colorectal, gastric, lung, urothelial, and hepatic cancers, as well as in several other malignancies (14). A prospective study reported that PNI not only predicts postoperative complications in colorectal cancer but may also confer a protective effect against their occurrence (15). Furthermore, PNI has been established as a significant predictor of short-term postoperative complications following radical resection of colorectal tumors. Sugawara et al. found that lower PNI levels were strongly associated with advanced patient age and more advanced pathological staging, findings that are consistent with the present study (16). Similarly, Luo et al. identified low PNI as an independent risk factor for overall survival and recurrence-free survival following gastrectomy (17). Migita et al. also reported that changes in PNI levels were independent predictors of disease-specific and overall survival in patients with GC following radical resection (18). Patients with decreased PNI were more prone to distant metastasis than those with increased or stable PNI.

### 4.3 Comparison of combined Fib and PNI scoring versus the predictive ability of individual Fib and PNI indicators

This study evaluated the prognostic value of preoperative Fib and PNI in predicting long-term survival in patients undergoing radical resection for resectable GC. Analysis of clinicopathological characteristics demonstrated that elevated Fib levels and low PNI values were significantly associated with advanced tumor stage, decreased five-year survival rates, and shorter overall survival durations. Both biomarkers were identified as potential prognostic risk factors in this patient population.

Given the limitations of Fib and PNI as standalone indicators, a combined scoring system (F-PNI) was investigated to improve predictive accuracy. ROC curve analysis was used to assess the prognostic performance of each marker. The AUC was 0.587 for Fib and 0.592 for PNI. When integrated into the composite F-PNI score, the AUC increased to 0.633, which was significantly higher than that of either biomarker alone (*p* < 0.05). This finding indicates that the F-PNI score provides superior discriminatory ability in predicting overall mortality following radical gastrectomy.

Although the individual AUC values fell within the moderate range (0.60 – 0.65), their combined use improved prognostic accuracy. This suggests that single biomarkers may not sufficiently capture the multifactorial nature of GC progression and prognosis. Despite the moderate AUC, Fib, PNI, and F-PNI were all included in both univariate and multivariate Cox regression analyses. Among these, only the F-PNI score emerged as an independent prognostic factor influencing five-year overall survival.

The findings of this study confirm that the combined F-PNI score demonstrates superior prognostic performance compared to Fib and PNI when used independently. Higher F-PNI scores were associated with more advanced tumor stages, lower five-year survival rates, and poorer overall outcomes, underscoring its relevance as a prognostic biomarker in patients undergoing radical gastrectomy for resectable GC. As an easily obtainable and cost-effective parameter derived from routine preoperative testing, the F-PNI score holds significant clinical value for risk stratification and individualized treatment planning. However, the absence of external validation and the lack of sensitivity analyses represent limitations of this study. Further prospective, multicenter investigations are warranted to validate the generalizability and clinical applicability of the F-PNI scoring system.

### 4.4 Preoperative optimization strategies for high F-PNI scores

There is increasing international recognition of the importance of comprehensive management strategies for GC, which now encompass not only neoadjuvant therapy, surgical resection, and postoperative adjuvant chemoradiotherapy, but also interventions aimed at improving nutritional and inflammatory status (19–22). Nutritional support, in particular, has emerged as a key component of cancer care. Evidence suggests that effective nutritional interventions can significantly reduce the incidence of treatment-related complications and improve both immune and nutritional function in patients with GC (23).

For patients with a low PNI, targeted nutritional support plays a pivotal role in improving clinical outcomes. In those with preserved gastrointestinal function, the use of enteral nutrition formulas enriched with immunomodulatory components is recommended (24). Daily protein intake should be increased to 1.5–2.0 g/kg of body weight, with additional supplementation of ω-3 polyunsaturated fatty acids and glutamine. ω-3 fatty acids may indirectly reduce Fib levels by inhibiting the release of pro-inflammatory cytokines (25), while glutamine contributes to the protection of the intestinal mucosal barrier and mitigates inflammatory responses associated with endotoxin translocation (26).

In patients with impaired oral intake or malabsorption, parenteral nutrition should be initiated. This includes supplementation with amino acids, fat emulsions, and vitamins via central venous access to address hypoalbuminemia and overall nutritional deficiencies. In cases of elevated Fib levels, nutritional support should be complemented by anti-inflammatory and anticoagulant therapies. Low-molecular-weight heparin is recommended due to its dual function in inhibiting coagulation factors and modulating inflammatory responses, thereby reducing Fib synthesis (27).

For patients with markedly elevated Fib concentrations (>5 g/L), short-term use of low-dose antifibrinolytic agents such as batroxobin may be considered (28). Additionally, non-steroidal anti-inflammatory drugs may be administered to attenuate systemic inflammation and suppress Fib production by inhibiting cyclooxygenase-2 activity (29).

Importantly, these preoperative interventions should be closely integrated into the broader oncologic treatment plan. During the course of nutritional, anti-inflammatory, and anticoagulant support, regular monitoring of PNI and Fib levels is essential. A multidisciplinary, synergistic approach to regulating these parameters within an optimal range prior to surgery may reduce the incidence of postoperative complications and improve long-term outcomes in patients with GC.

## 5 Conclusion

The combined F-PNI score was identified as an independent prognostic factor for long-term survival in patients undergoing radical gastrectomy for resectable GC. Compared with Fib and PNI assessed individually, the F-PNI score demonstrated superior predictive performance. Given its accessibility, cost-effectiveness, and association with key pathological features and survival outcomes, the F-PNI score holds significant clinical utility as a preoperative risk stratification tool in the management of GC.

## Data Availability

The raw data supporting the conclusions of this article will be made available by the authors, without undue reservation.
